# The effect of CAG repeats length on differences in hirsutism among healthy Israeli women of different ethnicities

**DOI:** 10.1371/journal.pone.0195046

**Published:** 2018-03-27

**Authors:** Naomi Weintrob, Ori Eyal, Meital Slakman, Anat Segev Becker, Galit Israeli, Ofra Kalter-Leibovici, Shay Ben-Shachar

**Affiliations:** 1 Pediatric Endocrinology and Diabetes Unit, Dana-Dwek Children's Hospital, Tel Aviv Sourasky Medical Center, Tel Aviv, Israel; 2 Sackler Faculty of Medicine, Tel Aviv University, Tel Aviv, Israel; 3 The Gertner Institute for Epidemiology & Health Policy Research, Tel-Hashomer, Israel; 4 Genetic Institute, Tel Aviv Sourasky Medical Center, Tel Aviv, Israel; Universite Clermont Auvergne, FRANCE

## Abstract

**Purpose:**

Variations in the degree of hirsutism among women of different ethnic backgrounds may stem from multiple etiologies. Shorter length of the polymorphic CAG repeats of the *androgen receptor* (*AR*) gene may be associated with increased activity of the receptor leading to hirsutism. We hypothesized that there are ethnic differences in the degree of hirsutism that is unrelated to androgen levels among Israeli women, and that the CAG repeats length may contribute to these differences. Anti-androgenic therapies, such as spironolactone, could be suggested if a shorter CAG repeats length is found to affect the difference in the degree of hirsutism between the ethnic groups.

**Methods:**

Healthy Israeli Jewish women aged 18–45 years of Ashkenazi and non-Ashkenazi origin were invited to participate. Hirsutism was assessed using the simplified Ferriman–Gallwey (sFG) score, and serum total testosterone levels were measured as well. The CAG repeats length was determined by PCR. Methylation-sensitive methods were used to detect the fractional activity of each allele, and the weighted mean was calculated for the CAG repeats length.

**Results:**

One-hundred and eight women were recruited (49 Ashkenazi and 59 non-Ashkenazi). The Ashkenazi women had a significantly lower degree of hirsutism (*P*<0.01), lower mean BMI (*P* = 0.003), total testosterone levels (*P* = 0.017), and longer weighted bi-allelic CAG repeats mean (*P* = 0.015) compared to non-Ashkenazi women. For the group as a whole, there was a significant negative correlation between the number of CAG repeats in the *AR* gene and the sFG score, while the number of repeats was not related to testosterone levels. Stepwise logistic regression revealed that ethnic origin and the CAG repeats length were the strongest factors affecting hirsutism (*P*<0.001, *P* = 0.03, respectively).

**Conclusions:**

There is a significant difference in the degree of hirsutism between Ashkenazi and non-Ashkenazi women in Israel that is partially explained by CAG repeats length.

## Introduction

The androgen receptor (AR) is the key protein controlling cellular androgen sensitivity, thus making it a promising candidate modifier of the degree of hirsutism. The *AR* gene is located on the X chromosome[1] and it contains a highly polymorphic region with a variable number of CAG repeats (CAG)_n_, which encodes a polyglutamine tract in the N-terminal transactivation domain of the receptor.[2] The number of CAG repeats normally ranges between 9 to 38 (mean range between 21 to 23), and it varies with ethnicity.[3–5]. Previous *in vitro* studies have shown a negative association between AR transactivation and CAG repeats length,[2, 6] implying that an *AR* with a shorter CAG repeats polymorphism has higher activity. Some epidemiological studies have associated a shorter CAG repeats with hyperandrogenic syndromes, such as precocious pubarche or polycystic ovary syndrome (PCOS).[7–11] In addition, the *AR* gene is subjected to X inactivation in females and, given its common polymorphism, it is the one usually used to detect the degree of X chromosome inactivation in women.[12] The pattern of gene inactivation may affect the phenotype as well, since it determines which of the 2 alleles has higher functional effect in a given cell.

Hirsutism, defined as the growth of male pattern terminal hair, is one of the most frequent complaints of post-menarchal females and affects ~4–7% of premenopausal women.[13, 14] The degree of hirsutism was traditionally assessed by the Ferriman-Gallwey score which involves 11 body areas.[15] It was changed to the modified Ferriman-Gallwey score. which involves 9 body areas.[16] Since both scoring methods involve a full physical examination and were considered invasive by many women, a simplified noninvasive method for assessing the degree of hirsutism was studied and validated against the modified Ferriman-Gallwey score.[17] That method includes checking the extent of terminal body and facial hair growth in 3 body areas, the chin, and the upper and lower abdomen. It proved to be highly correlated with the modified Ferriman-Gallwey score and was termed the simplified Ferriman-Gallwey score (sFG).[17]

The prevalence of hirsutism has been described as being different among various ethnic groups[18, 19] independent of androgen levels. In a previous study on Jewish women with non-classic congenital adrenal hyperplasia (NCCAH),[5] we found that women of Ashkenazi Jewish (AJ) origin had a significant longer CAG repeats length compared to women of non-Ashkenazi Jewish (NAJ) origin. In this study, we aimed to further investigate whether there is an ethnic-related difference in the degree of hirsutism among healthy AJ and NAJ women in Israel, and whether such a difference is associated with various factors, including the CAG repeats length. Since it has been suggested that skewed inactivation of the X-chromosome carrying the longer CAG repeats might explain the degree of hirsutism in non-hyperandrogenic women,[20] we also compared the degree of skewed inactivation between the 2 ethnic groups. Anti-androgenic therapies, such as spironolactone, can be suggested when a shorter CAG repeats length is found to affect the difference in the degree of hirsutism between the ethnic groups.

## Materials and methods

### Study population

The study was performed in the Pediatric Endocrinology Unit at the Dana-Dwek Children’s Hospital and the Genetic Institute at Tel-Aviv Medical Center. The study protocol was approved by the medical ethics committee of Tel Aviv Sourasky Medical Center (Helsinki #0038-13-TLV) and the national genetic ethics committee of the Ministry of Health (Ministry of Health# 920130094). Premenopausal healthy Jewish women aged 18–45 years of Ashkenazi, Sephardic or middle-Eastern origin were eligible. Women with known hyperandrogenic syndromes, such as PCOS[21], NCCAH[5] or laboratory findings of androgen excess,[22] women receiving hormonal treatment including hormonal contraceptives or receiving epilation cosmetic treatment during the last 3 months, and women with infertility were excluded. Each woman was given a written explanation on the study, signed an informed consent form, and was asked to fill in a short questionnaire on her menstrual cycles, fertility history and use of cosmetic treatment. The degree of hirsutism was assessed by one observer (M.S.) using the sFG score.[17] Blood for total testosterone was drawn on day 2 to 5 of the menstrual cycle. The serum testosterone level was determined by chemiluminescence (Rosh, Cobas A 411). Normal levels of total testosterone range between 0.2 to 0.6 ng/ml in our lab.

### Molecular analyses of the androgen receptor

DNA extraction from peripheral blood leukocytes was performed by using a salting out technique according to a standard protocol. The CAG repeats length and the X inactivation pattern were determined by PCR as previously described.[5, 23] The weighted bi-allelic CAG repeats measurement (WBAM) was calculated for each subject based on the relative expression of each allele in the blood lymphocytes (as described in detail previously [5]).

### Statistical analysis

Statistical analysis was performed using the BMDP software.[24] Descriptive statistics are given as mean and standard deviation (SD) for continuous variables and frequency distribution for categorical variables. Associations between CAG repeats length and degree of hirsutism were assessed using Spearman's correlation. Discrete variables were compared using the Chi-square test or Fisher’s exact test, as appropriate. Comparisons between continuous variables were performed using one-way analysis of variance (ANOVA). Equality of variance was tested using Levene's test, and the equality of means test (Welch or Brown-Forsythe) if it were found to be significant. Whenever there were any significant differences, pairwise t-tests were employed in order to examine each difference separately, using the Bonferroni correction for multiple comparisons. To evaluate the relative association between various factors and the degree of hirsutism, we applied a logistic regression model with the sFG as a dependent variable and body mass index (BMI), ethnic origin, testosterone levels and CAG repeats length as independent variables. We applied this model again excluding ethnicity from the independent variables in order to assess the relative contribution of the CAG repeats length to the degree of hirsutism. A *P* value of ≤ 0.05 was considered significant.

## Results

One-hundred and eight premenopausal Jewish women were recruited (median age 37.5 years, range 18–45 years). Forty-nine women were in the AJ group and 59 were in the NAJ group. Their mean±SD weight was 62.3±11.1 kg, mean height 164.0±6.2 cm, and mean BMI 23.0±4.1kg/m^2^. The mean age at menarche for the entire cohort was 12.9±1.5 years, and the mean number of pregnancies was 2.2±1.8. Age at study, age at menarche and the number of live births were similar for the 2 study groups (Table 1). There was no difference in the mean number of pregnancies between the two groups (2 per AJ woman vs. 2.3 per NAJ woman, *P* = 0.26), but the proportion of women reporting spontaneous abortion was significantly higher in the NAJ group compared to the AJ group (21% vs. 7%, respectively, *P* = 0.003). AJ women had a significantly lower mean BMI and testosterone values compared to NAJ women (*P* = 0.003, *P* = 0.017, respectively) (Table 1).

**Table 1:** Demographic, clinical, biochemical and genetics characterization of health Ashkenazy and non-Ashkenazi women.

Characteristic	Ashkenazi (49)	Non-Ashkenazi (59)	*P*-value
Age, y (mean ± SD)	8.81 ± 37.1	37.0 ± 9.21	0.99
Menarche, y (mean ± SD)	12.8 ± 1.61	12.9 ± 1.41	0.84
History of severe acne, n	3 (6.1%)	8 (13.6%)	0.2
Pregnancies, n	98	137	0.26
Spontaneous abortions, n	7 (7%)	29 (21%)	**0.003**
BMI	21.7 ± 3.61	24.0 ± 4.31	**0.003**
Testosterone (ng/ml)*	0.15 ± 0.13	0.22 ± 0.17	**0.017**
sFG score	1.2 ± 1.6	3.2 ± 2.6	**<0.001**
sFG score ≥3	16 (33%)	36 (61%)	**0.004**
History of epilation laser treatment, n	13 (26.5%)	28 (47.5%)	**0.03**
WBAM CAG repeats	22.2 ± 2.4	21.1 ± 2.3	**0.015**

SD, Standard deviation; BMI, Body max index; ng/dL, Nanogram/deciliter; sFG, Simplified Ferriman-Gallwey score; WBAM, Weighted mean of the bi-allelic measurement. The values in **bold** are significant.

*Normal range 0.2–0.6 ng/ml in our lab

**Fig 1:**
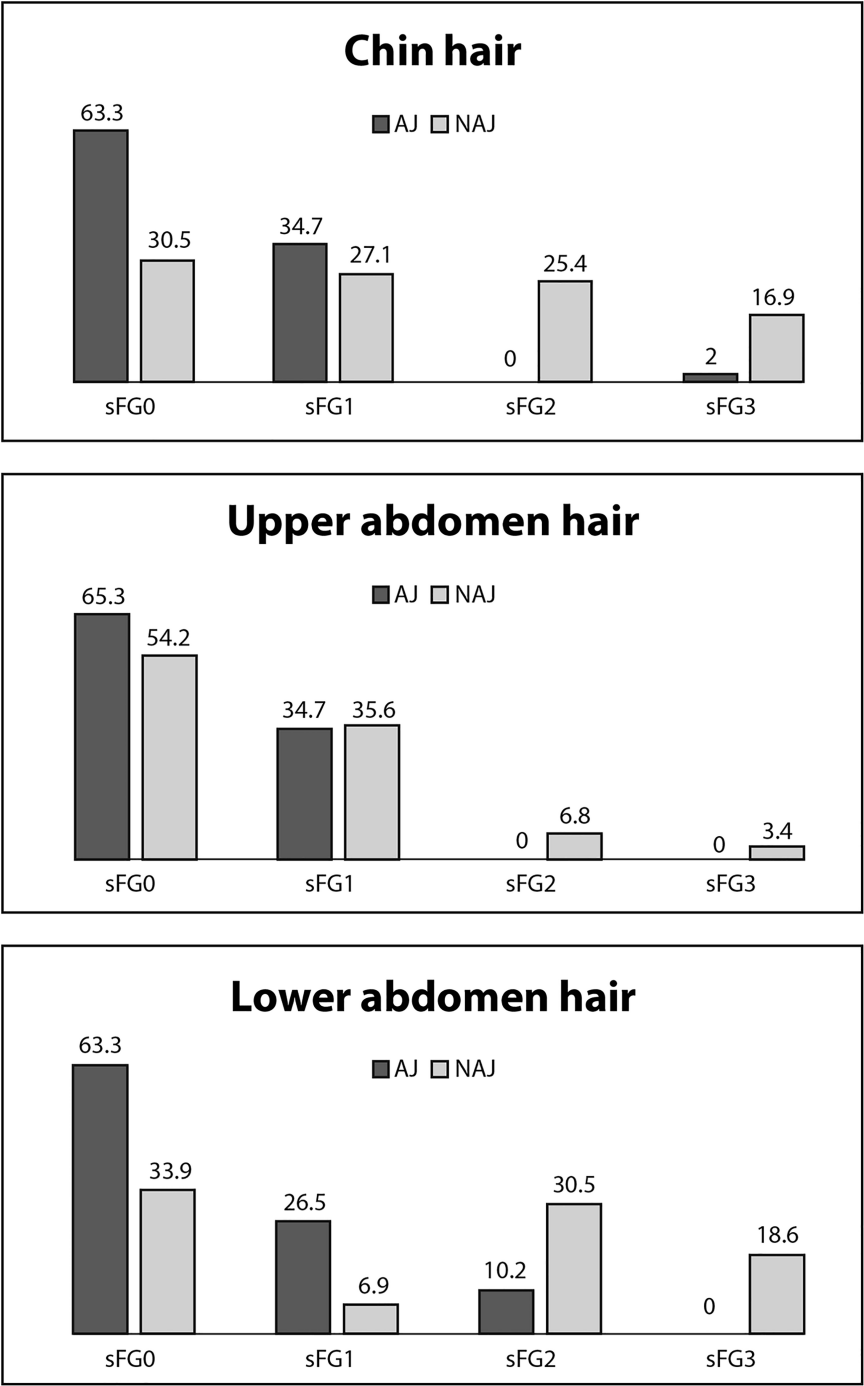
Simplified Ferriman–Gallwey (sFG) score of different body areas among Ashkenazi and non-Ashkenazi women.

The degree of hirsutism as measured by the sFG score was significantly higher among the NAJ women compared to the AJ women (3.2±2.6 vs. 1.2±1.6, respectively, *P*<0.001) (Table 1 and Fig 1). Defining hirsutism as an sFG score ≥3,[17] there were 53/108 (49.1%) women with hirsutism, of whom 16/49 (32.7%) were AJ women and 36/59 (61.0%) were NAJ women (*P* = 0.004) (Table 1). Furthermore, the proportion of women without any degree of terminal hair (an sFG score of zero) was more than twice as high among AJ as in NAJ women (57.1% vs. 21%, respectively, *P*<0.001). A history of cosmetic laser treatment for excess hair growth (performed more than 3 months before study recruitment) was more prevalent among the NAJ women (47.5% vs 26.5% for the AJ women, *P* = 0.03).

The WBAM of CAG repeats was significantly longer in the AJ group compared to the NAJ group (*P* = 0.015) (Table 1). The rate of skewed X inactivation was similar between the 2 groups. Twelve of the 47 AJ women (25.5%) with informative data (i.e., the existence of 2 alleles with a different size) and 18 of the 54 informative NAJ women (22.2%) had skewed X-inactivation (*P* = 0.73).

Testing for correlations between the different variables studied in the whole cohort yielded a positive correlation between the sFG score and testosterone levels (R = 0.31, *P* = 0.01), and a negative correlation between the sFG score and the WBAM CAG repeats (R = -0.31, *P* = 0.01). In addition, there was a positive correlation between the BMI and the sFG score (r = 0.23, *P* = 0.017), and a negative correlation between the BMI and WBAM CAG repeats (r = -0.24,*P* = 0.013). There was a trend for a positive correlation between BMI and testosterone level (r = 0.17, *P* = 0.09) and no correlation between the testosterone levels and WBAM CAG repeats.

Applying a stepwise logistic regression model to the entire group to determine factors significantly associated with the degree of hirsutism revealed that both ethnic origin (OR 2.96, 95%CI 1.32–6.34, *p* = 0.007) and WBAM CAG (OR = 0.83, 95%CI 0.69–1.00, p = 0.049) were significant while total testosterone and BMI were not. When applying the same model and excluding ethnicity, WBAM CAG repeats remained the only significant factor affecting the degree of hirsutism (OR 0.77, 95%CI 0.62–1.00, *p* = 0.004), while BMI and testosterone levels made no additional contribution.

## Discussion

The results of this study demonstrated that NAJ women had significantly higher sFG scores and rates of hirsutism (sFG score ≥3) compared to AJ women. The 32.7% rate of hirsutism among the AJ women in our study was similar the rate of 31.5% reported for a large North American female cohort.[17] In contrast, the rate of hirsutism in the NAJ group was significantly higher (61%). Reports on ethnic-related differences in the rates of hirsutism are inconsistent. For example, no differences between Caucasians and African Americans in the United States were detected by DeUgarte et al.[13] In contrast, Americans of Far Eastern origin showed no hirsutism whatsoever compared with a 40% rate of hirsutism among American Caucasians,[25] and women from central Europe tended to have a higher degree of hirsutism compared to women from Southern Europe.[22] These ethnic differences suggest a genetic contribution to hirsutism.

Based on the significant affect of testosterone levels [22] and BMI[26, 27] on hirsutism, we hypothesized that the ethnic difference in hirsutism score between our 2 study groups may be associated with those contributing factors. Indeed, the NAJ women had higher levels of total testosterone (within the normal range) and higher BMIs compared to the AJ women. However, those differences lost significance when we applied a stepwise logistic regression model, leaving only ethnicity and CAG repeats length as significant factors for predicting hirsutism, and supporting the role of genetics in the etiology of normo-androgenic hirsutism.

One likely mechanism for idiopathic hirsutism is an increase in the sensitivity of the hair follicles to androgens, possibly because of alternation in the androgen signal transduction system in the target tissue level. While most genetic factors associated with hirsutism are yet unknown, a negative correlation between CAG repeats length and hirsutism had been detected by Chamberlain et al.[2] Some studies showed a negative association between CAG repeats length and the prevalence of higher androgenic states, such as precocious pubarche,[7] PCOS[8, 10] or more severe clinical hyperandrogenism, such as hirsutism in subjects with PCOS.[28] In contrast, in a recent meta-analysis and systematic review of studies correlating CAG repeats length with the rate of PCOS, one-half of the analyzed studies found such a correlation while the others did not.[11] Indeed, applying the multiple logistic regression model while excluding ethnicity CAG repeats length was the strongest modifier of the sFG score in the current work. The role of ethnic origin observed in our study raises the possibility that such an effect exists in other populations as well. It might be, therefore, that the conflicting results on the correlations between CAG repeats length and hyper-androgenic conditions[11] are related to the different ethnic groups that had been included in that meta-analysis.

Our earlier study on Jewish subjects with non-classic 21 hydroxylase deficiency demonstrated that CAG repeats length contributes to the clinical diversity of the phenotype.[5] Interestingly, we also found that Ashkenazi Jewish females with non-classic 21 hydroxylase deficiency had a longer CAG repeats length compared to the non-Ashkenazi Jewish females.

This study confirmed a significant difference in WBAM CAG repeats length between healthy AJ and NAJ women. The AJ women in our study had a longer repeat length in concordance with their decreased level of hirsutism. To our best knowledge, this is the first study to show a difference in CAG repeats length and its possible association to the degree of hirsutism among different ethnic groups in normo-androgenic healthy females.

Interestingly, the rate of miscarriages was higher among the women in our NAJ group. It is still unresolved whether women with hyperandrogenic states have an increased risk of miscarriage compared with non-hyperandrogenic controls. A large Australian study[29] demonstrated that the miscarriage rate was more frequent in women with PCOS than in controls. We have recently shown a significantly higher rate of spontaneous miscarriages among women with non-classical congenital adrenal hyperplasia compared with the general Israeli population.[30] However, other studies have not shown comparable results.[31] In addition, there is an association between BMI and recurrent spontaneous abortions.[32] It is possible that the higher testosterone level (albeit within the normal range), higher BMIs and shorter CAG repeats (which may increase the androgen signal transduction at the target tissue level) contributed to the higher rate of spontaneous abortions in the NAJ women.

As expected, there was a positive correlation between sFG scores and testosterone levels.[22, 26, 27] The negative correlation between sFG scores and the WBAM CAG repeats together with the absence of any correlation between testosterone levels and CAG repeats length suggest that CAG repeats length contributed, to some degree, to the hirsutism difference between the 2 ethnic groups in the current study.

Our study has some limitations that bear mention. First, a hyperandrogenic state, such as PCOS or NCCAH, was ruled out based solely on self-reports and total testosterone levels. Second, ethnic origin was also based on self-reports as well. However, such reporting is considered reliable based on the immigration of the majority of the Jewish population to Israel having taken place after 1948. Third, recruitment of the participants was based on volunteering sample which might cause a selection bias towards more hirsute women, although that applies to both study groups.

In conclusion, the findings of the current study revealed a significant difference in the degree of hirsutism and miscarriages between Ashkenazi Jewish women and non-Ashkenazi Jewish women in Israel, and that it might be partially explained by CAG repeats length.
