# Correction: The effect of CAG repeats length on differences in hirsutism among healthy Israeli women of different ethnicities

**DOI:** 10.1371/journal.pone.0203181

**Published:** 2018-08-24

**Authors:** Naomi Weintrob, Ori Eyal, Meital Slakman, Anat Segev Becker, Maya Ish-Shalom, Galit Israeli, Ofra Kalter-Leibovici, Shay Ben-Shachar

Dr. Maya Ish-Shalom should be included in the author byline. She should be listed as the fifth author, and her affiliations are 2 Sackler Faculty of Medicine, Tel Aviv University, Tel Aviv, Israel and The Institute of Endocrinology, Metabolism and Hypertension, Tel Aviv Sourasky Medical Center, Israel. The contributions of this author are as follows: Investigation, Resources.

The correct citation is: Weintrob N, Eyal O, Slakman M, Segev Becker A, Ish-Shalom M, Israeli G, et al. (2018) The effect of CAG repeats length on differences in hirsutism among healthy Israeli women of different ethnicities. PLoS ONE 13(3): e0195046. https://doi.org/10.1371/journal.pone.0195046
